# Exploring adverse parent-child relationships from the perspective of convicted child murderers: A South African qualitative study

**DOI:** 10.1371/journal.pone.0196772

**Published:** 2018-05-23

**Authors:** Bianca Dekel, Naeemah Abrahams, Michelle Andipatin

**Affiliations:** 1 Gender and Health Research Unit, South African Medical Research Council, Cape Town, Western Cape, South Africa; 2 University of the Western Cape, Cape Town, Western Cape, South Africa; 3 School of Public Health, University of the Western Cape, Cape Town, South Africa; TNO, NETHERLANDS

## Abstract

Child homicide is the most extreme form of violence against children. Within South Africa, children face the highest risk of homicide by parents/caregivers. It is suggested that prolonged exposure to adverse relationships with one’s own parents may be linked to committing child homicide as it may lead to psychological damage and disturb neurological functioning. This paper explores the adverse parent-child relationships of 22 men and women incarcerated for the murder of either a biological child, a stepchild or a child in their care and draws on 49 in-depth interviews with these participants. We illustrate that traumatic parent-child experiences in the form of absent parents, neglect and abuse have a profound impact on establishing unhealthy attachment styles and emphasize the importance of early adverse parent-child bonds in setting the tone for future bonds as adults. The pathway to adopting an adverse attachment with one’s own child is argued to be influenced by these early traumatic emotional experiences within the home. This study highlights the need to acknowledge the impact that adverse parent-child experiences have on the formation of violent forms of parental behavior. It is imperative to reduce children’s emotional vulnerabilities by implementing strategies to strengthen current parenting practices, to promote the development of less violent parent-child relationships and to work towards resolving parents’ experiences of trauma in reducing child homicide.

## Introduction

Child homicide by a parent is an unfathomable crime for many. Although it is an age-old practice [1], a paucity of research has been conducted in developing settings. The 2014 report on violence against children published by UNICEF found the under five year age group had the second largest number of homicides among children, followed by the 15–19 year age group [2]. The South African national child homicide study found a similar pattern [3]. Among the under five year group, more than half (53.2%) were neonates (defined as 0–28 days old) and infants (74.4%) (defined as under 1 years of age), giving a neonaticide rate of 19.6 per 100,000 live births and an infanticide rate of 28.4 per 100,000 live births [4]. The study reported among the highest rates for neonaticide and infanticide, surpassed only by the estimate for Dar es Salaam (27.7 per 100,000 live births) [5], and are much higher than those reported in developed settings [6]. However, the South African study did not limit the perpetrators to parents given the South Africa context of childcare, with many children not raised by biological parents [7]. The South African child homicide study showed however, that a parent was the perpetrator in the majority of cases [4].

Child homicide is poorly understood in South Africa and not much is known about the psychosocial processes underlying parent’s homicidal acts. Men and women with histories of child abuse are more likely than non-abused men and women to engage in violence toward children [8]. Thus, understanding the role of poor and abusive parent-child relationships in childhood (and adulthood) and its impact on violence within the adults’ relationship with their own children is important. Attachment theory and epigenetics provides a framework to begin the engagement to understand child homicide. Attachment theory suggests the parent-child relationship plays a role in the development of mental representations of self and others, which provides the foundation for relationships and emotional regulation [9]. Loving parenting promotes secure attachments: Most of these children become securely bonded and emotionally balanced adults/parents. Harsh/rejecting parenting promotes adverse attachments and the development of an inability to regulate and interpret their own feelings and that of others, as the caregiver is a source of both fear and comfort, which has important implications for the development of personality disorders. Interactions with one’s child (whether this is an interaction with a biological or stepchild) are largely influenced by the caregiver’s own attachment style and is affected by whether or not the individual has resolved early traumatic experiences. A parent’s adverse attachment style with his/her children can result in him/her exhibiting poor parenting skills. He/she may battle to tolerate feelings that have reawakened painful childhood memories of abuse from his/her own parent. Therefore, he/she may act out aggressively toward his/her child [10].

Epigenetics has emerged as a theory to aid in the understanding of violence in later life. Epigenetic research has opened the biological to environmental/social influences and to the impact of early experience on the later use of violence [11]. Social/environmental factors play an instrumental role in human biology and social/environmental insults can leave long-term indelible scars on the body and brain. For example, epigenetics demonstrates that there is a direct link between maternal care and neurological development/cellular modifications of the child and thus, proposes that the first 1000 days of a child’s life from conception are critical in terms of the effects of early experience on neurological development. The sustained effects of these cellular modifications form the basis for the developmental origins of vulnerability to violence. Therefore, early traumatic life experiences become embedded in the “memory” of a person and it is thus, recognized that the interaction between environmental and biological factors is imperative to consider in the development of crime and violence [12].

Many children in South Africa have difficult childhoods, for example, numerous children do not live in the same home as their parents and experience a sequence of different caregivers [7]. This is largely a result of factors, including labor migration, poverty, housing and educational opportunities. It is common for relatives (mainly grandmothers) to play a role in childrearing, which is mainly the responsibility of women [13]. In developed settings, it is not uncommon for women who commit child homicide to cite feeling overburdened as the main motive [14]. In South Africa, a significant number of pregnancies occur in families where support during and after pregnancy rests almost solely on the mother and overall in 2010, 53% of pregnant women were single. For many of these women, pregnancies increase their financial and health-related vulnerabilities and adversely affects their life chances and that of their children, as many mothers and children live in poverty [15]. In developed settings, women have cited financial difficulties as the primary motive for child homicide [16] and indeed, in South Africa in 2014, 30% of children (5.5 million) lived in households where no adults were working and the unemployment rate was slightly higher for women (28%) than for men (23%) [13].

Poverty is a societal risk factor for child abuse [15]. Reducing poverty (and thus, the stress on families) is a vital aspect of reducing child abuse, as is equipping parents/caregivers with the skills for non-violent forms of discipline. By the time young people in South Africa are 15–17 years old, many would have experienced abuse and neglect. A recent national South African study collected information from 9730 adolescents between the ages of 15 and 17 years old, stratified between households and schools and found one in five young people reported having experienced some form of sexual abuse in their lifetimes and one in three young people reported physical abuse. A total of 16.1% of young people experienced emotional abuse and one fifth of the young people reported experiencing child neglect at some point in their life. The study also found gender differences in the experiences of abuse, with girls experiencing more abuse, neglect and bullying. These young people are more likely to develop mental and physical health problems. These, in turn, undermine their capacity to succeed in life (at school and at work) and to maintain healthy relationships. These preventable issues cost South Africa greatly, both in terms of the costs of treating these problems, and in lost economic productivity [17].

This paper explores the parent-child relationships between the parents/caregivers convicted of child homicide and their own parents. Child homicide definitions are delineated to refer to different victim ages. Neonaticide is defined as the killing of a neonate on the day of its birth (first 24 hours) by his or her parent(s) [18] and infanticide as the murder of a child in the first year of his or her life by a parent(s) while filicide refer to the killing of a child by a parent [19]. This study focuses on parents and caregivers (defined herein as biological, step and de facto parents) who have been convicted of the death of a child in their care.

Many children in South Africa endure childhoods characterized by adversity. However, the difference for these participants is located in the emotional experiences of adverse fathering and mothering. The parent-child relationship is the first relationship in the infant’s life and according to attachment theory and epigenetics, sets the tone for the bond between the adult child and his/her own child. Due to the established importance of that first relationship, it is vital that we start our inquiry into the lives of men and women who killed their children by considering the stories they told about their own parents. This study was part of a PhD project where the overall purpose was to explore the participants’ childhood, adolescence, and adulthood experiences, their relationships with their children and partners, as well as what they perceived to have led to the homicidal act.

## Methods

Ethical approval was obtained from the Humanities and Social Sciences Research and Ethics Committee at the University of the Western Cape and the South African Medical Research Council. Approval to conduct research within correctional centers was provided by the Department of Correctional Services. Interviews were conducted with individuals incarcerated for the death of a child. A sample of 22 convicted men and women (8 men and 14 women) (see Table 1) were recruited from five correctional centers, based within the Western Cape Province of South Africa. Two centers were medium centers, two centers housed both medium and maximum offenders, and finally, one center was a maximum center. Three correctional centers housed males only and two centers housed both males and females.

**Table 1:** Description of perpetrators and victims.

Perpetrator Pseudo-name	Relationship to victim	Perpetrator age at time of interview	Victim age at time of death	Victim’s manner of death	Number of interviews conducted
Michelle	Caregiver	23	2 years old	Fatal child abuse	2
Thandi	Mother	31	3 years old	Fatal child abuse	1
Deidre	Mother	22	1 year and 2 months	Neglect	2
Latifa	Stepmother	36	5 years old	Fatal child abuse	2
Winnie	Mother	20	1 week old	Buried alive	1
Nicole	Mother	34	2 years old	Fatal child abuse	2
Ryan	Caregiver	32	2 years old	Fatal child abuse	2
Adam	Father	45	12 years old	Strangulation	3
Michael	Father	53	21 months old, 5 years old, and 16 years old	Firearm	3
Zolu	Father	33	2 years old	Buried alive	2
Abigail	Stepmother	34	6 months old	Sharp injury (knife)	1
Lauren	Mother	28	Newborn	Sharp injury (knife)	2
Zubeidah	Mother	34	2 years old	Smothering	3
Christelle	Mother	36	6 months old	Smothering	3
Cayleigh	Stepmother	32	7 years old	Smothering	3
Patricia	Mother	32	1 years old	Poisoning	2
Jennifer	Mother	29	3 months old	Fatal child abuse	3
Nelly	Mother	41	9 years old	Set child on fire	2
James	Stepfather	36	5 years old	Fatal child abuse	2
Jamaal	Father	38	8 years old	Strangulation	3
Sipho	Father	30	2 years old	Fatal child abuse	3
Howard	Stepfather	33	2 years old	Sharp injury (knife)	2

Purposive and snowballing sampling was used to recruit participants. Initially recruitment began using purposive sampling through utilizing each correctional center’s psychology department. Correctional center psychologists identified men and women (parents/caregivers) who were incarcerated for the death of a child and asked each offender whether he/she would be willing to participate. If he/she agreed, a suitable date and time would be organized for us to meet. Fourteen participants were identified using purposive sampling. Furthermore, eight participants were identified using snowball sampling. Once interviews commenced, offenders voluntarily provided additional names of offenders known to them. All of the prospective participants agreed to participate.

During each interview, the first author was present, as well as the participant and the relevant translator; no member (warden) ever sat inside the room during the interview, in order to ensure confidentiality. Participants had a choice as to their preferred language for the interviews. An Afrikaans and an isiXhosa translator were available and accompanied the first author if needed. After the interviews were conducted, the first author transcribed all English interviews and some Afrikaans interviews, with assistance from the Afrikaans translator, while the isiXhosa translator transcribed the isiXhosa interviews. Quality checks were done by the first author who conducted random crosschecking of the recorded interviews against the transcribed interviews. Informed consent procedures were followed and the aim of the study as well as all ethical procedures (risks and benefits, anonymity, confidentiality) and the recording of the interviews were explained to participants. To maintain anonymity we use pseudo names in the paper. A reward was not provided to those who agreed to participate as this study was conducted in a correctional center setting. We recognized that support for these participants was vital and thus, prior to each interview we asked each correctional center psychologist whether he/she would be willing to meet with the offender after the interview, if he/she felt this was needed. All correctional center psychologists agreed and five participants were referred to psychologists. Psychological support was also arranged for the first author, who conducted the interviews.

The first author conducted individual, semi-structured interviews, which enabled interviews to be flexible and allowed for the probing of areas of interest. A total of 49 interviews were conducted, and each interview ranged between one to two hours. A scope of enquiry was developed based on the literature reviewed, and used to guide the interviews, which allowed the agenda to be flexible although partially directed by the interview schedule. The first interview explored the participant’s background: their childhood and adolescent experiences, in particular their relationship with their father and mother. Examples of questions asked include, “Tell me about your childhood life”; “Tell me about your mother/father”; “Do you have siblings?”; “What was it like being a teenager?”. The participants spent a great deal of the interviews speaking about their relationships with their own parents. They also touched on the beginning of their intimate relationships, and for some, teenage pregnancy. The initial interview steered the second interview, which focused on their relationships with their own children, their desire to be a parent as well as the factors surrounding the actual death of the child. Examples of questions asked include, “How did you meet your spouse?”; “How was your and his/her relationship”; “Did you want to be a father/mother?”; “What do you think contributed to your child’s death?”. The third interview entailed follow up questions and provided participants with an opportunity to elaborate or provide further detail on the information provided in interviews one and two. Atlas T.I version 8.0 (Berlin, Germany) assisted in data management, which was performed according to the principles of grounded theory [20]. During open coding, audio-recordings, interview transcripts, participant observation and field notes were reviewed with the aim of examining the text for thoughts, ideas, meaning and consequently assigning them codes. Emphasis was placed on allowing concepts to emerge naturally without forcing them into predefined categories. Categories were divided into sub-categories, which was important as 108 codes were created. Trimming down of the codes was done with the assistance of the second author, thereby concluding the first stage with 54 codes. Together, a single category was identified (axial coding) as the central phenomenon of interest. This selection was made based on the category most extensively discussed by participants (i.e. the code with the highest frequency), which was then positioned as a central feature, around which, other categories were related and a storyline was constructed. To illustrate this storyline, discriminate sampling was used, which refers to selecting certain participant quotes, which are able to maximize opportunities for verifying the storyline. Part of engaging in selective coding entailed making use of memos, containing ideas about codes and their interconnections. These memos allowed for comparisons between data and codes to find similarities and differences. The storyline was further validated by searching for relevant literature pertaining to categories. Member checking was performed with participants to iron out ideas and reach consensus, and the storyline was discussed with co-authors. Lastly, core categories were then organized into the research paper. Quotes used herein are illustrative of the codes selected.

## Findings

The study includes 22 participants (14 females and 8 males) responsible for the death of 25 children. They were fathers (4), mothers (10), stepfathers (3), stepmothers (3) and caregivers (2). Their ages ranged between 17 and 43 at the time of the murder, and between 20 and 53 at the time of the interviews. Participants were racially categorized as African (7), Colored (11) and White (4) (using the Apartheid racial classification system). Two participants (one father and one stepfather) killed more than one child (three and two children respectively) and the remaining participants killed one child each. The victims ranged in age from newborn to 16 years old. The manner of death was fatal child abuse, i.e., abuse or negligent treatment resulting in the child’s death, in the context of a relationship of responsibility and care. These are listed in Table 1. They received sentences ranging from 8 months to life imprisonment. Twenty participants were raised in communities where socio economic hardships were common and experiences reported included violence, poor policing, unemployment, alcoholism, domestic violence, gangs and increased crime. Of the 22 participants, six had prior convictions.

For the participants, talking about their parents was emotionally difficult and it often elicited tears. One of the effects of adverse childhood experiences, coupled with adverse attachment styles to parental figures, involves a risk of developing mental illness, and a retreat from empathy, which is one of the foundations of emotional intelligence and moral judgement [21].

## Abusive parent-child relationships

### Physical abuse: “*she stabbed me with a knife*”

Eleven participants reported physical abuse by a parent during childhood/adolescence. This abuse began at early ages, for example, from “*one year and four months*” (Nelly) and “*from six years*” (Patricia), and endured for many years. Abuse by fathers were reported as Zubeidah explains: “*My daddy*, *he hit me with a spade already*. *I got kicked*, *yoh*, *it was bad the beatings*”. James was convicted of the murder of his stepson, aged five years old, whom he physically beat to death. As a child, James was frequently hit with: *“a fan belt of a car”* around the age of eight; an indication of the cycle of abuse. Attachment theory posits that people tend to parent in line with the context of their own prior experiences [22]. For example, a common belief in the legitimacy of harsh discipline is mediated (in part) by the connection between the experience of punitive discipline in childhood and the perpetration of it as an adult [23]. Some participants (“*for me its normal*, *its the way I was raised*”—James) reiterated that they disciplined their children in line with how they themselves were harshly disciplined. Epigenetics emphasizes that parental care can be transmitted from one generation to the next [24]. James further explained that the beatings were largely because of his “*plain naughtiness*” which he ascribed to the lack of love and guidance he received while growing up, which was due to his father’s alcoholism and the loss of his mother through death.

Physical abuse was not only perpetrated by biological parents, as Deidre explains: *“Me and my stepmother we didn’t get along… I was 12 years old when she stabbed me with a knife”*. She was raised by her father as her mother abandoned her at the age of eight: “*My stepmother was jealous because when my father gets his money*, *he will wait for me*, *to come from school so we can go to town*. *He didn’t like wait for her*”. Abuse experienced during early adolescence (12 years old) may lead to difficulty coping with school demands (Deidre left school shortly after the stabbing), socially withdrawn behavior (“*I was not like a social person*. *I was always on my own*. *I was never with friends*”), and low levels of empathy (“*I didn’t feel bad*”—she was previously arrested for robbery).

Eleven participants reported abuse by a mother. For example, Zubeidah was convicted of the murder of her two year old son and described occasions where her mother physically abused her: *“My mommy used to throw me with boiling water and yoh she’d beat me”*. She explained that the abuse was a result of her “*naughtiness*” which was displayed through “*stealing and lying*” and which she explains, “*was just to seek attention”*. This behavior as well as the abuse endured until she was 23 years old: “*The stealing started when I was six years old*… *The beatings stopped when I got married at the age of 23*, *because I couldn’t take it anymore*. *That was my scapegoat*. *To get married and to get out of the house*, *because I couldn’t handle my mommy’s abuse anymore*”. She explained that her mother was cold (“*don’t get that love and affection and hugging and kissing*”), which was largely a result of Zubeidah’s rebellious behavior, while her father “*was always at work*”. The lack of love and attention resulted in disobedient behavior in her younger years (“*the stealing started at the age of six*”) and in adulthood manifested in seeking attention from men (she had numerous, consecutive boyfriends), making her vulnerable to Intimate Partner Violence (IPV). It is common for children who do not receive enough attention, to act out in ways they mistakenly believe will provide them with the positive attention they crave. In hindsight, she acknowledges that her behavior served to drive her mother further away: “*Now I realize that wasn’t the right way to get her attention because when I did something wrong then she got angry and wouldn’t want anything to do with me again*, *but back then I didn’t realize*”. An angry response may have been rewarding, as attachment theory posits that disconnection, isolation, and loneliness becomes unbearable and thus, any response appears to be better than no response [25]. Furthermore, many participants described similar childhood experiences. A 42 year old father who killed his daughter explains: “*She* (mother) *beat us*, *very much*, *very bad*”. Jennifer also recounted her experiences of being abused by her mother, which began from roughly the age of six. She said: “*My mom hitted me one day with a plate over my head …*.*She would hit me with a broomstick or a mop*, *whatever she could get in her two hands*”. The intergenerational cycle of abuse was evident in Jennifer’s account as she had asked her mother why she had abused her: “*She said*, “*I want you to feel my hurt and my pain and my suffering that I went through in my life*””. At the age of 21, Jennifer was found guilty of fatal child abuse of her three month old daughter. Patterns of interaction formed during interactions with one’s own parents, tend to influence the style of interactions that individuals will eventually have with children in their care [26].

### Sexual abuse: *“to my father I wasn’t his child; to him I was a wife”*

Nine of the women we interviewed experienced sexual abuse, yet Cayleigh was the only participant who reported sexual abuse at the hands of a parent. She was raised by both parents until the age of 13 when her father passed away (which is also when the abuse ended). After her father’s passing, her mother abandoned her and she lived with different family members, strangers and in foster homes: “*The last day that I saw my mother was the day of my father’s funeral*”. She could not remember when the sexual abuse began, but knew that it endured “*for as long as I can remember*”. Cayleigh further explained: “*To my father I wasn’t his child; to him I was a wife… My father will like say to me like I must go on my knees and I must lick him like a lollypop”*.

Cayleigh explained: “*If I refuse to do what he asks me to do with him*, *he will beat me…I was never allowed to cry*, *no matter how painful it was*. *Otherwise*, *he will beat me up badly… Because he said crying is for babies*”. She mentioned that she often felt “*scared*” as a child as she was frequently confused and frightened because she could not anticipate what to expect next (“*As a child I was always confused*, *I never knew when or what to expect*”). It was evident that the sexual abuse was still extremely painful to speak about and she often cried during the interviews. At the age of 23, Cayleigh murdered her seven year old stepdaughter whom she smothered with a pillow. Cayleigh was pregnant at the time with her son. Childhood sexual abuse may carve a path through many adult lives of those who survive it and may leave a legacy of this abuse.

### Un-protective parents: *“For her to stand and to watch what he’s doing to me and not doing anything was worse than the abuse”*

Participants reminisced on how they felt about one of their parents not protecting them from abuse from another parent/stepparent. Deidre, who was an only child, was physically abused by her stepmother and recalled the lack of protection offered by her father: *“My dad didn’t do anything when he sees she used to beat me up*. *It made me hate my dad*. *He see what this woman is doing to me every time she wants to beat me up and you doing nothing to stop her*!*”* Cayleigh, who was sexually and physically abused by her father, also spoke of a lack of protection from her mother: *“My mother will stand and watch him*, *what he’s busy doing with me*, *and she will do nothing*. *For her to stand and to watch what he’s doing to me and not doing anything was worse than the abuse”*. Epigenetic research proposes that young children do not have the means to curtail distress they may feel and therefore, the child relies on its mother to comfort him/her. An un-protective mother does not buffer the child’s stress [21, 24]. Attachment theory asserts that the vulnerability of having an inadequately protective mother in one’s maternal role development provides insight into formative experiences that shaped the consciousness of her as a mother [22]. Thus, as Calyeigh moved into the maternal role, these experiences informed how she integrated caregiving responsibilities of motherhood. Given this framework, perhaps it should not be surprising that she was unable to protect the child she killed from her own anger.

Cayleigh reflected on why she thinks her mother did not protect her: “*He did abuse her a lot and maybe she was scared of him*”. Years later, she found herself in an abusive relationship, yet she still maintained that: “*It seems to me that my mother did like being abused*. *Because she would not do anything or take me and run away or go to her family*”. She expressed more disdain towards her mother for not protecting her than towards her abusive father, which may be tied to her strong belief that it is “*a mother’s role to protect her child*”, yet she herself was convicted of murdering her stepdaughter.

Cayleigh’s father impregnated her at the age of eleven: *“My mother made me to have an abortion from that man*, *saying to the Doctor that I’m having sex with older men and I said to the Doctor*, *“she’s lying*. *It is my father”*. She explained that the Doctor, “*didn’t wanted to listen to me and it was painful*. *I was asking myself*, *“will there ever be someone in my life that will listen to me*?*””*. Cayleigh was not only subjected to a lack of protection from her mother, but also from institutionalized protective services, alluding to the failure to protect occurring on multiple levels attesting to the notion that the act of killing a child is a result of a plethora of contributing factors in a complex social context of multiple layers of deprivation. On another occasion: “*I ran out of the house because I couldn’t handle the abuse anymore and they* (neighbors) *will see like blood on me or they will hear noises and they will come and check”*. As a result, the neighbors phoned the police: “*We were standing in the police station and my mother said to this policeman*, *“I’m sorry sir*, *I can’t let my husband go to prison for this bitch*””. Cayleigh’s narrative illustrates that there were opportunities for protective services to intercept and assist her, but her mother prevented this from happening as her mother “*said to the policeman that I’m naughty and I don’t want to listen*. *So he was just reacting like a father and the policeman believed my mother*… *A mother is someone who is supposed to be there for her children*, *look after them*, *protect them*”.

The offenders we spoke to also sought protection from verbal/emotional abuse, as Jamaal explained: “*My stepmom will say*, *“you will become nothing in life”*. *I was about 12 years old*. *It was almost like a tape recorder playing… She abused me verbally… I had to stand up for myself*”. Jamaal and Deidre’s narratives (mentioned earlier) allude to stepmother jealousy of stepchildren. Twenty of the 22 participants’ parents permitted them to be abused by others and they themselves beat their own children and often left their children behind, hoping to begin a new life elsewhere.

## Abandonment and rejection

### Absent parents and idealizing mothers: *“I don’t know how it feels to have a mom”*

Among the 22 participants, more than half (15 participants) reported being physically abandoned by a parent. Some of the participants were raised by both parents and then were abandoned later on and raised in single parent households and some were raised by single parents only. A few participants grew up without either of their biological parents in their lives and were raised by grandparents.

Deidre was an only child and was abandoned by her mother at the age of eight: *“My mother walked away from home and she never came back*. *My world fell apart when she left”*. Her father raised her thereafter. Ryan’s older brother passed away when Ryan was a teenager. Shortly thereafter his parents’ divorced, resulting in Ryan’s father becoming physically absent from his life. Ryan experienced two great losses simultaneously: “*I was cross at my father because he is the person*, *which boys are supposed to look up to… Then he just disappeared … I feel disappointed and sad*”. In contrast, Latifa, who was convicted of the death of her five year old stepson, says that her biological father was absent since birth: “*My biological father*, *he just carries the name “father”*, *but he is nothing*”. Latifa was the only participant who said it did not bother her that her biological father was absent, as her mother was an emotionally secure parent to her.

### Idealizing mothers

The male participants placed more importance on their mothers and her absence was considered a greater loss than the absence of a father. Their narratives highlight the societal construction of the role of mothers and the idealizing of mothers. Yet, consistently, each participant mentioned that their mothers hurt them the most emotionally: “*It’s my mother*. *She hurt me very very much*” (Patricia).

Sipho, who physically beat his son to death with a brick when he was 24 years old, grew up without knowing both parents. He explained that: “*To grow up without a mom is very hard*. *It was difficult to see other children with their mothers*, *painful*. *I’m hurt; I don’t know how it feels to have a mom*”. His grandmother raised him until the age of 20, when she passed away, and Sipho was forced to live with his father, who was a stranger. Many of the participants did not live consistently in the same home. An unstable living environment has been associated with the inability to manage stress (for example, in times of stressful childrearing). Being abandoned by his parents resulted in the development of an adverse attachment, which influenced his feelings of being abandoned once again when his grandmother passed away. Individuals who are adversely attached react to periods of separation with feelings of abandonment, jealousy, and aggression. Sipho beat his son to death and beat his then girlfriend upon suspecting that she was being unfaithful. Adverse attachment is also associated with substance abuse and Sipho was intoxicated at the time of the crime.

Sipho also idealized the mother of the child he murdered, who is the same girlfriend he is incarcerated for attempting to kill, along with the killing of his son. Sipho’s girlfriend had ‘fallen’ from grace minutes before the murder of his son and the attempted murder of her. He spoke about feeling ‘belittled’ and ‘humiliated’ by his girlfriend, which seemed to emasculate him, as evidenced in previous research with South African homicide offenders [27]. For Sipho, this idealized, once ‘perfect’ woman, was now flawed due to her alleged adultery, which was not fitting. Sipho engaged in internal splitting towards his mother and his girlfriend, as individuals are perceived as all good or all bad [28]. Splitting is related to a history of trauma and is conceptualized as an unconscious process, which separates contradictory feelings and representations of ‘good’ and ‘bad’, and is thought to develop to protect a positive representation of, for instance, his mother and girlfriend. Sipho explained that he believed a mother would be able to provide more guidance than a father would. Sipho’s belief, which is shared by Howard, as illustrated later on, highlights their disappointment in father figures. Howard (who was convicted of the double murder of his two year old twin stepdaughters) was abandoned by his mother at the age of six years old. Howard was subsequently raised by his father, who was frequently absent from home. His mother, just like Deidre’s mother, walked away from their home, never to return: *“I missed a mother in my life and it was very difficult because I didn’t have a mother… It was very hard and it is still hard for me”*.

Although his father was in his life, he noted that: “*There was nobody to guide me*”. Both Sipho and Howard placed greater emphasis on their mothers: “*It’s very important to have a mother because every child needs a mother’s love*. *A father can’t give that love*” (Howard). Both men believed that mothers are able to listen better and are the ones who welcome their children to speak to them, as Howard explains: “*Your mother will understand you better than your father*”.

## Parent-child bond (between perpetrators of child homicide and their own children)

Childhood trauma can affect how parents bond with their children. From an epigenetic perspective, parental betrayal may result in more affectionless, un-empathic interpersonal behavior, which in turn may increase the likelihood of violent behavior [12] and according to attachment theory, vulnerabilities can foster certain triggers which increase the risk for abuse to be transmitted to the next generation [9]. Yet, some of the participants conveyed that they were able to form satisfactory bonds with the children they killed. Whether their accounts of having happy relationships with their children are a true or exaggerated representation is unknown. This may reflect the idealization following the death of a loved one. Patricia explained that her daughter “*was my best friend*” and Jamaal explained that the times spent with the son he killed “*was the happiest days of my life*”.

During the interviews, participants spoke of their children with little affect. James explained that he does not feel “*love in my heart*” for his daughters who are still alive, even stating that “*I don’t want to be around kids*. *I don’t want them in my life*, *even my own*”. Christelle maintained that “*he’s my son*, *but I don’t have that kind of bond with him*” and when referring to the daily caregiving responsibilities said: “*I didn’t do it because I wanted to*, *but because I had to do it*”. It is not uncommon for parents to enact the same distancing with their own children, reflecting their struggles to bond with their children and reflecting their own possible fear of abandonment. It seems as though these unresolved issues led some to an avoidance of intimacy, and for others, these issues bled into their relationships, in the form of violence.

## Poor parental support systems in adulthood

The rejection the participants experienced was not limited to their childhoods and the abandonment continued into adulthood for sixteen of the participants. Prior to committing their crimes, many participants turned to their parents to receive help and in many instances were turned away. For example, Zubeidah who was convicted of her son’s murder, was in an abusive relationship with his father. Similarly, Christelle, who was convicted of the murder of her son and boyfriend, was also in an abusive relationship with her boyfriend. Seven of the women who discussed the men in their lives indicated they were beaten by them. Zubeidah purposely married her husband in an attempt to get out of her parent’s abusive home, yet in hindsight says: “*I am ever so sorry that I got married*”. There is a commonality of childhood abuse and IPV: Women may be conditioned to accept violence from those they love, and their limited ability to distance themselves from abusive relationships seems to lead to their entanglement in criminal activities. Both Zubeidah and Christelle had left their partners and had taken their sons multiple times to their parents’ home and would always eventually go back to their partners. Again, both women had attempted to move back into their parents’ homes shortly before the murders. Zubeidah maintained that her parents were “*fed up*” because “*they know I always go back to my husband*”. Likewise, Christelle explained that her parents had “*had enough*” of her leaving her boyfriend, moving into her parents’ home, and then reuniting with her boyfriend again. On the day of Zubeidah’s son’s death, she decided to go home to her parents’ house in an attempt to leave her husband for the last time: “*I took Taahir and I went to my mother*. *She said “no”*. *I went to my father and he listened to what my mother said*”. She killed her son, Taahir, that same day out of sheer desperation, having nowhere to go and not being able to provide for him. She maintained the lack of support, coupled with the cumulative adverse childhood/adolescent experiences drove her to commit the crime. Similarly, Christelle maintained that “*It’s because of my father over the years; he put us out of the house… If I had a better support system maybe I would have done that*, *like to take my baby and go and live with my parents*”. Although these participants’ accounts may be a true representation, we cannot ignore that it appears that they are attempting to shift the blame and in so doing, to not accept responsibility for their crimes.

Patricia had been independent since she was kicked out of her parent’s home eight years prior: *“They did chase me away*, *when I was pregnant with that first baby”*. She lived alone with her two daughters until she contracted HIV. Patricia was convicted for the murder of her eldest daughter and for the attempted murder of her youngest daughter. She lost her job and was unable to provide for her daughters. Just like Zubeidah, Patricia was turned away in the months preceding the murders: *“I think them rejecting me*, *that’s what put me here*. *If I was having a loving mother and father*, *a home*, *where I can share everything with them*, *I think it was going to be easy for me to go to them and say “I’m sick now”*. *I was not going to suffer alone*. *I wasn’t going to be here in prison”*. Parenting is at best a complex process and factors such as poverty, job and family instability add immeasurably to the inherent difficulties. Since Patricia’s motive was financial, she believes that had her parents allowed her and her daughters to stay with them; her youngest daughter would still be alive. Patricia had a tumultuous relationship with her mother since her earliest memory: “*She wasn’t like a mother to me … my whole life she is making my heart sore… she is always shouting and swearing at me… she damaged me inside… I never even have a hug from her or a kiss from my mother*”. Patricia’s narrative resembles Jennifer’s narrative of an intergenerational cycle of abuse noted earlier. Patricia explains: “*I asked her why she treat me like that all the years and she said to me*, *“I remember the things that I did to you but it was because of the way I was raised up”*. *She was raised by her uncle and his wife and they was treating her differently than with their children*, *so that’s why she’s doing it to me*, *now you see it’s like history repeating itself*”.

## Emotional rejection: *“what I needed was father love”*

Among the 22 participants, twenty experienced emotional rejection by a parent. A parent’s psychological unavailability is a form of child maltreatment, which plays a role in the development of violent behavior. Jennifer was raped at the age of six by an older stepbrother and explained that, “*For me it was difficult because I couldn’t talk to anyone*”. She had an emotionally and physically abusive relationship with her mother and felt her mother was unapproachable. Attachment theory suggests that for mothers who have experienced relationships of unavailability and fear, the responsibility for the well-being of their own infant can be particularly overwhelming. For instance, the new mother’s response to her infant’s distress can be impeded by her own (unconscious) memories of experiences of fear and unavailability from her own mother. The relationship carries both sides of a possibly polarized internal working model as the mother may experience herself as both the angry and unavailable parent and may thus, feel she is caught between opposing fears [29].

Jennifer proceeded to explain that: *“The rejection*. *The rejection*. *It is worse than the rape*. *I’m struggling still with that”*. Jennifer struggled with contradictory images of a ‘loving’ and ‘abusive’ mother: “*She used to abuse me a lot*, *but she’d also give me love*. *There was two sides to her and that was confusing for me*. *I don’t understand my mom*”. Such parenting behavior leads to the development of an inability to interpret one’s own feelings and that of others, as the parent is a source of both fear and comfort, which could have important implications for the development of mental illness [9]. This type of parenting behavior also normalizes the occurrence of love and abuse, teaching Jennifer from a young age that it is ‘normal’ for love and abuse to co-exist. Years later, she found herself in an abusive relationship with the father of her baby. It is likely that Jennifer’s mother suffered from a mental illness (“*There was two sides to her*”), which is susceptible to intergenerational transmission, according to epigenetics [30]. Indeed Jennifer stated that she suffers from depression, an indication of the transmission of mental illness.

The emotionally cold and absent type of parenting behavior displayed by participant’s parents, left them feeling unloved and ‘searching for love’. Jamaal, in referring to his eight year old son whom he killed, said: “*The love that I gave my son*, *was the love my father didn’t give me*”- throughout his childhood and adolescent years. Howard explained that: “*When I was younger*, *like a child and also a teenager*, *my father was always busy*. *He didn’t have time for me*”. For both of these men, the perceived rejection by their fathers and the lack of father love meant that they searched for male affirmation outside the home, within gangs, leading to an identification with violent models of masculinities.

Deidre said that when she turned nine: “*My father didn’t know it’s my birthday and I was crying*. *I missed my mother and my dad didn’t even know it was my birthday*”. The absence of a father figure had a similar impact on the women. At the age of 18, Deidre started dating a man (Norbit) who was ten years older than her and who had previous convictions: “*What I needed was father love and I couldn’t get it from my father so Norbit was there for m*e”. Likewise, Patricia explained she met a man at the age of 20 and “*he came to me and then he say he love me and that was the first day I hear the words “I love you*””. Patricia’s narrative resembles the notion that all abused parents do not continue the cycle of violence: “*I was telling myself that I want to be a good mother to that child*, *not a mother like my mother was*. *I want her to have a good life*. *She mustn’t hear the words “I love you” on the streets*, *there by the boys*. *She must hear it from me”*. Although this was the daughter she was convicted of killing, she was aware of the intergenerational transmission of abuse and tried her best to not fall into the same cycle, and in many ways, she escaped the cycle in terms of daily abuse, yet ultimately could not prevent herself from being part of it entirely.

## Emotional rejection due to drugs and alcohol

Ten participants were raised by parents/caregivers addicted to substances. Addiction largely entails an emotional abandonment. Ryan’s father was an alcoholic: “*As soon as I see or hear his name*, *I think alcohol*”. Once Sipho’s father entered into his life, he was physically present, yet emotionally unavailable because he was: *“All the time drunk*, *drunk*, *drunk”*. As a result, Sipho’s father *“disappoint me”*. Deidre, who lived with her father explained that: *“My dad just wanted to drink and have his own life doing his own thing… Me and my father did not have a relationship… I needed somebody to talk to and to be there for me when I ask a question and my dad wasn’t there”*. Nicole, imprisoned for the fatal child abuse of her two year old son, stated that: *“My mother was a drinker*. *She drinks all the time*. *If she had no drink*, *she would sleep the whole day*, *every day”*. Likewise, James stated, “*my father is an alcoholic”*. According to the intergenerational cycle of substance abuse, it is not surprising that many of these offenders also abused substances as adults, with substance abuse often playing a role in the crime they committed. The abuse of alcohol and drugs by the participant’s parents often resulted in a role reversal.

## Parentification: “I couldn’t play outside like other children”

Due to the rejection and abandonment experienced by these participants, it is anticipated that a lack of parental guidance would be a part of this. Michelle stated, with reference to her father, that: “*I left school at Grade 7; he didn’t even tell me like*, *no*, *I must go back to school*”. In many instances, there was a great deal of role reversal, whereby as children, these participants, were tasked with the responsibility of looking after their parents, which was largely a result of their parent’s substance abuse, as a then 14 year old Michelle explains: *“If my daddy work on a Saturday*, *he will drink afterwards*. *Then I will stress and think*, *“where’s this man*?*” and go and fetch him*. *So for me it was a very stressing thing to look after a big person*. *Because they rob people*, *they kill people outside*, *you see*? *Now for me I have to sit*, *I’m a child but I have to sit and worry about him but his big”*.

James was placed in a similar situation as a child: *“My father is an alcoholic*. *The people always asked*, *“Where is James*? *You must go and fetch your father because he is drunk and falling around””*. Deidre explained that instead of being able to play outside with other children, she was forced to take care of the home, since her mother abandoned her and her father was an alcoholic: “*I have to be the woman of the house… when I come from school… I have to do all the cleaning and I have to make food and I couldn’t play outside like other children*”.

## Ability to assume a paternal or maternal role: “*I don’t know how to be a mother to him*”

Attachment theory tends to conceptualize parental child homicide as the outcome of the intergenerational transmission of inadequate paternal/maternal role development, as it is suggested that fathers and mothers often develop a paternal or maternal identity, that somewhat mirrors that of their own parents [31]. Thus, childhood trauma can affect a parents’ ability to assume a paternal or maternal role, as evidenced in Cayleigh’s narrative: “*It’s difficult to give a child that you don’t have experience of*. *Like I need to be a parent to my son*, *a mother to him but I don’t know how to be a mother to him*, *because I never had that but now I must give him that*”. The physical and emotional abandonment these participants experienced meant they often admitted to not knowing what it means to be a parent, as simply illustrated by Winnie: “*I don’t know*”. Parenting is something that is largely learnt from one’s own parents. Yet, the majority of participants maintained they learnt parenting behavior from observing strangers. James explains: *“I actually have a lot of time to watch other people*, *how they are with their kids and stuff like that*. *I never have a father who can play with me and stuff like that and for me to see like okay that is how you must be with a kid”*.

## Wanting to be a ‘different’ parent: “*I was dreaming of being a different mother to how my mother was*”

Nineteen of the participants spoke about attempts to be ‘different’ fathers and mothers as compared to their parents. Patricia explains: “*I was dreaming of having a child of my own and to be a good mother to that child*, *a different mother*. *I was dreaming of being a different mother to how my mother was*”. The lack of a positive fathering or mothering experience may facilitate the longing to become a different parent, a wish to not repeat the pain that one has suffered as a child. This serves a functional purpose in assisting the adult child to reverse the earlier trauma of growing up in a harsh environment, while assisting him/her vicariously in fulfilling her/his own earlier unmet needs. For example, it is not uncommon for such parents to turn to their children for the love denied to them as children and indeed, some women attempted to fill this emptiness by having a child of their own: “*I did get the love I always wanted from my child*” (Patricia) and “*A woman needs a child for that love and affection*, *if someone else*, *like a parent doesn’t want to give it*, *then that child covers that*” (Zubeidah). Attachment theory states that, “when a woman with this background becomes a mother, there are times when instead of being ready to mother her child, she looks to the child to mother her” ([32], p. 86).

## A child’s unconditional love: “*I love my mommy… no matter she abuse me long time… my mommy don’t love me*, *but I love my mommy so much*”

Regardless of the abuse or abandonment these men and women experienced at the hands of their parents, they often cried the most when speaking about their parents and spoke of how they still yearn for their parent’s love. When children are abused or abandoned by a parent, they often internalize the experience, feel responsible, and blame themselves for causing the negative reaction by the attachment figure. Instead of integrating the good and bad aspects of the parent, the child splits off the bad aspects of the parent, so that the child can maintain a positive view of the parent. Thus, in spite of the trauma inflicted by their own parents, the men and women professed an undying loyalty to their parents.

The intertwining of a yearning for approval with a keen sense of vulnerability permeated their stories about their parents. Deidre, whose mother abandoned her at the age of eight, said that her mother hurt her the most emotionally, yet she wanted nothing more than to be reconnected with her. Jamaal was also protective of his mother, regardless of the inauspicious manner in which she had treated him throughout his life by abandoning him and failing to provide for him. He began providing for his mother when he became an adult, in ways she had been unable to do when he was a child. He went as far as resorting to crime to ensure that she never went hungry: *“I took care of my mother… My mother was an alcoholic*. *But I could understand for certain things she went through in her life*. *So I stood by her*. *I got into gangsterism and started selling drugs so that I could sort my mother out and put food on the table for her”*. Harsh parenting experiences, as exercised by Jamaal’s mother, increases the risk for externalizing violent behavior, as depicted in Jamaal’s narrative. Most of the men did not have an opportunity to complete school and therefore, were locked in poverty with restricted access to stable employment. With attainment of a provider role imperative for self-perceptions of masculine ‘success’ in South Africa, the lack of opportunities made achieving this difficult.

Zubeidah declared her love for her mother, even after being rejected and abused countless times: *“I love my mother*. *Irrespective of the way she treated me*, *I still love my mother”*. The participants continued to seek an emotional attachment to their parents and indeed, attachment theory suggests that we are programmed from birth to seek proximity to our parents [9]. Nelly shed the most tears when speaking about her mother and said: *“No matter my heart is sore*, *I love my mommy so much*. *No matter she abuse me since I was young*. *I try to accept that thing*, *that my mommy don’t love me*, *but I love my mommy so much”*. Even though their parents hurt them deeply—they were protective of them, which in many ways is expected given that parents tend to be a group of people we will always forgive. It may also be socially unacceptable not to love one’s parents. Acknowledging one’s parent as flawed could trigger a degree of humiliation, as children often feel ashamed of their parents’ failings. Nobody wants to admit that they lack something as fundamental as a parent’s love. Therefore, regardless of how badly these men and women were treated; they tended to love their parents unconditionally, and it is likely that they will continue to do so forever.

## Discussion

This paper explores childhood and adolescent trauma perpetrated by a group of parents and the perceived impact on attachment with their children and violent behavior as an adult. Many children in South Africa endure childhoods characterized by adversity. However, we argue that the difference for these men and women is located in the emotional experiences of adverse fathering and mothering resulting in the inability to develop a healthy internal model of a secure parent. The participants’ childhoods were marked by abusive, neglectful and absent parenting practices. Attachment theory and epigenetics have proven to be a fruitful theoretical lens from which to understand child homicide, as these theories conceptualize child homicide to be rooted within the intergenerational transmission of an inadequate parental attachment and role development [33]. The participants’ narratives appear to have many features in common with the adversely attached parental profile, which are indicative of a lack of secure attachments to parents. For example, their narratives suggest upbringings where experiences of fear were plentiful and accompanied by a lack of protection. Participants appeared aware of the hurt they felt while growing up and seemed committed to not inflicting the same fear, abuse or abandonment (i.e., wanting to be different parents). However, these participants lacked an inner representation available to them of a middle ground between frightening behavior and passivity and abandonment. Thus, with no internal models of balanced nurturance available to them, they seemed unable to parent in a healthier manner. However, childhood adversities are neither necessary nor sufficient to trigger the onset of violent behavior. Tied to this, the environment created by maltreating parents tends to not support the development of appropriate coping strategies and therefore, children raised in these conditions, may rely on aggression, dissociation, and avoidance to cope with adverse emotions [34]. As such, it seems as though participants (likely unconsciously) resorted to adverse strategies such as detaching from their emotions, and using drugs/alcohol, which may have aided the use of violence as a response to stressful emotional situations. Nevertheless, the narratives presented here offer a window into the intergenerational cycle of adverse parenting patterns [29].

Further, the reflective self tends to evolve in the context of a healthy infant-caregiver relationship [35], which participants likely lacked. Thus, it is possible that this resulted in an inability to understand their own as well as another’s’ state of mind, which entails the absence of the psychological capacity required to appropriately deal with adverse emotions (e.g. anger), and to regulate affective impulses, which possibly contributed to their perpetration of violence [36]. Their narratives point towards their own parents being unwilling/unable (for a variety of reasons) to understand their state of mind (e.g. when, as children, participants craved and needed love and attention), which likely meant they grew into adults who were also unable to do this. Having a parent who does not want to, or who cannot, take in a child’s state of mind, forces the adoption of another strategy; i.e. one that will not include accessing one’s own state of mind or that of others [37]. Thus, in studying attachment styles amongst violent offenders, it has been said that adverse attachment styles are “likely… linked with a developmental failure of empathy, which implies some degree of self-reflective function: it is hard to imagine the feelings of others if there is diminished capacity to think about one’s own feelings” ([38], p. 35). Parents who have a low reflective function, tend to lack the insight to comprehend that they are causing harm to their child and thus, are more likely to engage in harsh discipline practices [22]. It has been proposed that the self-reflective function is connected to the development of empathy and the failure of empathy is believed to be a vital contributing factor in the perpetration of violence [38].

The abuse and absence of their own parents were remarkable, and support previous findings on childhood maltreatment amongst child homicide perpetrators [39]. For many, the psychological impact of an absent or abusive parent was lasting, leaving them emotionally vulnerable. Indeed, attachment theory and epigenetics place emphasis on the notion of abuse/abandonment issues remaining ‘unresolved’ [40, 41], and we have shown that participants cried the most when speaking of their parents and the abuse/abandonment they experienced (alluding to unresolved issues). Years ago, the challenges adults with such histories face in freeing themselves psychologically from past traumatic experiences when they become parents were highlighted [34]. In a study conducted with mothers, it was found that childhood abuse and general maltreatment were associated with unresolved status and that such mothers were more likely to develop adverse attachment relationships with their children [42]. Because these issues remain unresolved, they may remain psychologically ‘alive’ in the mind of the individual and therefore, have the potential to influence behavior [38]. It has been postulated that when traumatic experiences have not been resolved, the parent’s memories and emotions associated with these experiences may be reactivated by the child’s behavior (e.g. an infant’s crying) and may provoke a dissociative state during which he/she engages in violent behavior with the child [43]. Research also demonstrates that parents, who have psychologically dealt with their own parents as rejecting, are not as likely to reject their children. This is in contrast with the parental functioning of parents who are yet to come to terms with the rejection experienced by their parents [44]. Thus, these unresolved issues may continue to have an on-going emotional and cognitive influence on the parent’s state of mind regarding childrearing [45, 46] and have the potential to “bleed out” ([47], p. 47) into relationships with children as this tends to adversely impact on their ability to form healthy relationships [31]. A limitation of this paper is inherent in the analysis of only the relationships with their own parents, as we acknowledge there are other factors within these men and women’s environments that further impacted on their ability to parent. Another limitation is that these are their subjective views, as we do not have the thoughts and opinions from family members to validate their experiences. Finally, a criticism of attachment theory is that it ignores aspects such as race, poverty and culture (which are imperative characteristics in South Africa), as it fails to consider the impact of these external factors on the relationship between parent and child [48]. History is not destiny, and whether parenthood becomes a repetition of the past in the present, or whether it becomes a time of renewal cannot be predicated solely based on the attachment narrative of the parental past [42]. There are additional external factors, which contribute to the development of violent behavior amongst parents. These external influences are analyzed in a separate paper. Nevertheless, when considered as part of a constellation of social and other factors, attachment theory contributes to our understanding of parental child homicide.

## Clinical implications and recommendations

There is no straightforward solution to this complex problem. Efforts to prevent child homicide should target parents/caregivers who have histories of experiencing child abuse/neglect, because their children are at increased risk of experiencing abuse/neglect. Fostering safe and nurturing relationships between parent and child, appears to be a crucial factor in breaking the intergenerational cycle of abuse [49]. Interventions aimed at improving parents/caregiver capacity to provide nurturing should simultaneously increase parents’ knowledge and ability to respond to factors plaguing resource poor settings, such as South Africa. Factors include widespread violence, substance abuse and high levels of poverty [50]. Home Visitation Programs (HVP’s) could improve the early bonding between parent/caregiver and child and reduce the risk of inappropriate punishment and abuse [51]. Vulnerable mothers need to be identified during pregnancy, and followed after the birth. Parents/caregivers need to be educated on non-violent parenting programs, which promotes positive parenting and discipline [51]. It is feasible for South Africa to utilize HVP’s and to add interventions to routine health services for pregnant women and new parents, as a parenting intervention to improve parent–child relationships [52]. HVP’s and center-based parenting skills training are of relevance in low-resource settings, where professional staffing is unlikely to be affordable at scale; health facilities may be inaccessible for many people, particularly in poorer areas and thus, the use of existing service delivery mechanisms (e.g., home visits) are more cost-effective. Thula Sana is a South African HVP that targets pregnant women and mothers of infants aged 0–2 years from low-resource communities and aims to promote sensitive, responsive engagement with their infants. An evaluation of this program found those mothers who participated in the home visits were significantly more sensitive and less intrusive in their interactions with their infants [53].

Efforts should be made to educate the public and professionals about child homicide risk factors [54]. This study has shown that professionals need to take abuse seriously and should ensure that complaints are thoroughly investigated. Child protection services need to act speedily in the investigation of reported cases to protect children from continued abuse and to prevent fatalities. Professionals require training to identify which children in the household are at risk; assess the needs of those children and the capacities of carers to create an environment that is safe and conducive to recovery. Police officers should be encouraged to use their powers under the Children’s Act to remove perpetrators when there are risks to children’s safety as assessed by social workers [55]. Child protective agencies should be encouraged to be receptive to accepting children into their care who are unwanted, even if no abuse has yet occurred. It is advised that clinic staff receive additional training in the Children’s Act Regulations, so as to enhance their ability to detect abuse and neglect and to make the reports and referrals in terms of the Act [51].

Men are to be encouraged to support their partners and active fathers are to be acknowledged. Fathers should also be screened (where possible) for stress factors such as poverty, in which the impact is amplified when these men have psychological issues stemming from childhood. As with mothers, depression in fathers has a detrimental effect on children’s development. Greater attention to fathers is needed [31]. Parenting classes, emotional support, and emergency numbers to call when parents/caregivers are overwhelmed can be helpful in preventing child homicides. Parents/caregivers need to be supported within their neighborhoods after the completion of programs and classes, if they are to maintain their resilience [56].

## Conclusion

Traumatic childhood experiences have a profound impact on parent-child bonds and highlight the importance of recognizing early adverse experiences on later adult violent behavior. The experience of poor/abusive parenting practices during childhood/adolescence hampered these men and women from attaining healthy attachments with their own children and thus, worked to continue the intergenerational cycle of abuse. This study has highlighted the need to acknowledge the impact these experiences have on an individual’s ability to adopt a paternal or maternal role. It is therefore, vital to reduce children’s emotional vulnerabilities by engaging in strategies to strengthen current parenting practices and to address the “ghosts in the nursery” ([42], p. 387) to promote the development of less violent parent-child relationships and to resolve parents’ traumatic experiences.

## Supporting information

S1 Data (DOCX)
