# Supplementary material for: Exploring adverse parent-child relationships from the perspective of convicted child murderers: A South African qualitative study
Source: PLoS One. 2018 May 23;13(5):e0196772. doi: 10.1371/journal.pone.0196772 (PMC5965825; doi:10.1371/journal.pone.0196772)
Supplement: S1 Data — (DOCX) [file pone.0196772.s001.docx]

**ZUBEIDAH:**

“*My daddy, he hit me with a spade already. I got kicked, yoh, it was bad the beatings*” … *“My mommy used to throw me with boiling water and yoh she'd beat me”….* “*naughtiness*” … “*stealing and lying*” … “*was just to seek attention”* … “*The stealing started when I was six years old... The beatings stopped when I got married at the age of 23, because I couldn't take it anymore. That was my scapegoat. To get married and to get out of the house, because I couldn't handle my mommy’s abuse anymore*”….“*don’t get that love and affection and hugging and kissing*”…“*was always at work*”…. “*the stealing started at the age of six*” … “*Now I realize that wasn’t the right way to get her attention because when I did something wrong then she got angry and wouldn’t want anything to do with me again, but back then I didn’t realize*”… “*I am ever so sorry that I got married*”. .. “*fed up*” because “*they know I always go back to my husband*”… “*I took Taahir and I went to my mother. She said “no”. I went to my father and he listened to what my mother said*”… “*A woman needs a child for that love and affection, if someone else, like a parent doesn’t want to give it, then that child covers that*” … *“I love my mother. Irrespective of the way she treated me, I still love my mother”.*

**JAMES**

“*love* *in my heart*” … “*I don’t want to be around kids. I don’t want them in my life, even my own*”… “*my father is an alcoholic”.*. *“a fan belt of a car”* ; “*for me its normal, its the way I was raised*” ; “*plain naughtiness*” … *“My father is an alcoholic. The people always asked, “Where is James? You must go and fetch your father because he is drunk and falling around”” …“I actually have a lot of time to watch other people, how they are with their kids and stuff like that. I never have a father who can play with me and stuff like that and for me to see like okay that is how you must be with a kid”.*

**CHRISTELLE**

“*he's my son, but I don't have that kind of bond with him*” … “*I didn’t do it because I wanted to, but because I had to do it*”… “*had enough*” …“*It's because of my father over the years; he put us out of the house… If I had a better support system maybe I would have done that, like to take my baby and go and live with my parents*”.

**CAYLEIGH**

“*The last day that I saw my mother was the day of my father’s funeral*”… “*for as long as I can remember*”… “*To my father I wasn’t his child; to him I was a wife…* *My father will like say to me like I must go on my knees and I must lick him like a lollypop”…* “*If I refuse to do what he asks me to do with him, he will beat me…I was never allowed to cry, no matter how painful it was. Otherwise, he will beat me up badly… Because he said crying is for babies*”… “*scared*” … “*As a child I was always confused, I never knew when or what to expect*”. *“My mother will stand and watch him, what he’s busy doing with me, and she will do nothing*. *For her to stand and to watch what he’s doing to me and not doing anything was worse than the abuse”. ..* “*He did abuse her a lot and maybe she was scared of him*”… “*It seems to me that my mother did like being abused. Because she would not do anything or take me and run away or go to her family*”…“*a mother’s role to protect her child*”… *“My mother made me to have an abortion from that man, saying to the Doctor that I’m having sex with older men and I said to the Doctor, “she’s lying. It is my father”.* … “*didn’t wanted to listen to me and it was painful. I was asking myself, “will there ever be someone in my life that will listen to me?””*. … “*I ran out of the house because I couldn’t handle the abuse anymore and they* *will see like blood on me or they will hear noises and they will come and check”*. … “*We were standing in the police station and my mother said to this policeman, “I’m sorry sir, I can’t let my husband go to prison for this bitch*””. … “*said to the policeman that I’m naughty and I don’t want to listen. So he was just reacting like a father and the policeman believed my mother*… *A mother is someone who is supposed to be there for her children, look after them, protect them*”. “*It’s difficult to give a child that you don’t have experience of. Like I need to be a parent to my son, a mother to him but I don’t know how to be a mother to him, because I never had that but now I must give him that*”.

**JENNIFER**

“*My mom hitted me one day with a plate over my head ….She would hit me with a broomstick or a mop, whatever she could get in her two hands*”. … “*She said,* “*I want you to feel my hurt and my pain and my suffering that I went through in my life*””. “*For me it was difficult because I couldn’t talk to anyone*”. … *“The rejection. The rejection. It is worse than the rape. I’m struggling still with that”.* ..“*She used to abuse me a lot, but she’d also give me love. There was two sides to her and that was confusing for me. I don’t understand my mom*”.

**JAMAAL**

“*My stepmom will say, “you will become nothing in life”. I was about 12 years old. It was almost like a tape recorder playing… She abused me verbally… I had to stand up for myself*”… “*was the happiest days of my life*”… “*The love that I gave my son, was the love my father didn’t give me*”…“*When I was younger, like a child and also a teenager*, *my father was always busy. He didn’t have time for me*”. .. *“I took care of my mother… My mother was an alcoholic. But I could understand for certain things she went through in her life. So I stood by her. I got into gangsterism and started selling drugs so that I could sort my mother out and put food on the table for her”.*

**NELLY**

“*one year and four months*” … *“No matter my heart is sore, I love my mommy so much. No matter she abuse me since I was young. I try to accept that thing, that my mommy don’t love me, but I love my mommy so much”.*

**DEIDRE**

*“My dad didn’t do anything when he sees she used to beat me up. It made me hate my dad. He see what this woman is doing to me every time she wants to beat me up and you doing nothing to stop her!”.* .. *“Me and my stepmother we didn’t get along… I was 12 years old when she stabbed me with a knife”…* “*My stepmother was jealous because when my father gets his money, he will wait for me, to come from school so we can go to town. He didn’t like wait for her*”…. “*I was not like a social person. I was always on my own. I was never with friends*” … “*I didn’t feel bad*”… *“My mother walked away from home and she never came back.* *My world fell apart when she left”…* “*My father didn’t know it’s my birthday and I was crying. I missed my mother and my dad didn’t even know it was my birthday*”. … “*What I needed was father love and I couldn’t get it from my father so Norbit was there for m*e”… *“My dad just wanted to drink and have his own life doing his own thing… Me and my father did not have a relationship… I needed somebody to talk to and to be there for me when I ask a question and my dad wasn’t there”…* “*I have to be the woman of the house… when I come from school… I have to do all the cleaning and I have to make food and I couldn’t play outside like other children*”.

**PATRICIA**

“*from six years*” … “*It’s my mother. She hurt me very very much*” … “*was my best friend*” … *“They did chase me away, when I was pregnant with that first baby”.* ..*“I think them rejecting me, that’s what put me here. If I was having a loving mother and father, a home, where I can share everything with them, I think it was going to be easy for me to go to them and say “I’m sick now”. I was not going to suffer alone. I wasn’t going to be here in prison”.* … “*She wasn’t like a mother to me … my whole life she is making my heart sore… she is always shouting and swearing at me… she damaged me inside… I never even have a hug from her or a kiss from my mother*”. … “*I asked her why she treat me like that all the years and she said to me, “I remember the things that I did to you but it was because of the way I was raised up”. She was raised by her uncle and his wife and they was treating her differently than with their children, so that’s why she’s doing it to me, now you see it’s like history repeating itself*”… “*he came to me and then he say he love me and that was the first day I hear the words “I love you*””…“*I was telling myself that I want to be a good mother to that child, not a mother like my mother was. I want her to have a good life. She mustn’t hear the words “I love you” on the streets, there by the boys. She must hear it from me”*. … “*I was dreaming of having a child of my own and to be a good mother to that child, a different mother. I was dreaming of being a different mother to how my mother was*”. … “*I did get the love I always wanted from my child*”.

**WINNIE**

“*I don’t know*”.

**RYAN**

“*I was cross at my father because he is the person, which boys are supposed to look up to… Then he just disappeared … I feel disappointed and sad*”…“*As soon as I see or hear his name, I think alcohol*”.

**SIPHO**

“*To grow up without a mom is very hard. It was difficult to see other children with their mothers, painful. I’m hurt; I don’t know how it feels to have a mom*”. .. *“All the time drunk, drunk, drunk” … “disappoint me”.*

**HOWARD**

*“I missed a mother in my life and it was very difficult because I didn't have a mother… It was very hard and it is still hard for me”…* “*There was nobody to guide me*”…“*It's very important to have a mother because every child needs a mother's love. A father can’t give that love*”… “*Your mother will understand you better than your father*”.

**ADAM**

“*She beat us, very much, very bad*”.

**LATIFA**

“*My biological father, he just carries the name “father”, but he is nothing*”.

**NICOLE**

*“My mother was a drinker. She drinks all the time. If she had no drink, she would sleep the whole day, every day”.*

**MICHELLE**

“*I left school at Grade 7; he didn’t even tell me like, no, I must go back to school*”. .. *“If my daddy work on a Saturday, he will drink afterwards. Then I will stress and think, “where’s this man?” and go and fetch him. So for me it was a very stressing thing to look after a big person. Because they rob people, they kill people outside, you see? Now for me I have to sit, I’m a child but I have to sit and worry about him but his big”.*
